# Multi-nutrient Optimization for Skeletal Advancement and Integrated Care in Pediatric Bone Health (MOSAIC-PBH): An Expert Consensus From India

**DOI:** 10.7759/cureus.117377

**Published:** 2026-10-03

**Authors:** Uday Pai, Ashwani Kumar, AV Ravishankar, Mahesh Sulakshane

**Affiliations:** 1 Pediatrics, Sai Kutir Clinic, Mumbai, IND; 2 Pediatric Medicine, Adesh Medical College And Hospital, Mohari, IND; 3 Pediatrics, M. K. Hospital, Chennai, IND; 4 Pediatrics, Kauvery Hospital, Chennai, IND; 5 Pediatrics and Child Health, Dr. Sulakshane Multispecialty Clinic, Pune, IND

**Keywords:** calcium, hidden hunger, magnesium, micronutrient deficiency, multi-nutrient approach, peak bone mass, pediatric bone health, vitamin d, zinc

## Abstract

Micronutrient deficiencies in children are common, often coexisting and subclinical, yet remain under-recognized. This expert consensus aims to provide an integrated framework for pediatric bone health, emphasizing multi-nutrient interactions and strategies to support adequate bone accrual and peak bone mass during growth. This consensus was developed using a modified Delphi methodology involving a multidisciplinary panel of 23 experts. A structured review of the literature was conducted to synthesize evidence on nutritional determinants, bone biology, and clinical management. Consensus statements were developed and subjected to a single-round anonymized Delphi voting process, with agreement defined as ≥75%. This expert consensus highlights the potential burden of dietary inadequacy and subclinical deficiency of key micronutrients, including vitamin D, calcium, magnesium, and zinc. Evidence supports a systems-based model of bone health, emphasizing the interdependence of structural minerals, regulatory cofactors, and hormonal pathways. A life-course approach was identified as essential, with critical windows during early life and adolescence. The panel recommends risk-based screening, comprehensive clinical assessment, and a context-specific approach to nutritional management that considers multiple nutrient and lifestyle factors for prevention and management. Practical guidance on formulation, dosing considerations, and implementation strategies is also provided. Pediatric bone health may benefit from a comprehensive, multi-nutrient and multi-mineral framework that complements assessment and management of individual nutrient deficiencies and is aligned with underlying physiology. Adoption of risk-based, life-course strategies and context-specific nutritional interventions may support skeletal health, while high-quality clinical studies are needed to establish the effects of multi-nutrient interventions on bone outcomes and fracture risk.

## Introduction and background

Pediatric bone health (PBH) is a key determinant of lifelong skeletal integrity, as bone accrual during childhood and adolescence directly influences the attainment of peak bone mass (PBM), a major predictor of osteoporosis and fracture risk in later life [1,2]. Approximately half of adult bone mass is accrued during adolescence, particularly during periods of rapid growth, making this a critical window for optimizing skeletal health [3]. Suboptimal bone accrual during these years is associated with increased fracture risk in childhood and reduced skeletal strength in adulthood [4]. Childhood fractures are common, with an annual incidence of 1.6-3.6%, and are increasingly recognized to be influenced not only by trauma but also by underlying bone quality [5]. Inadequate bone mass acquisition during growth may therefore result in lower PBM and greater susceptibility to osteoporosis and fragility fractures later in life [6-8].

These deficiencies may coexist and are associated with shifts toward nutritionally poor dietary patterns and reduced diet quality. The increasing consumption of energy-dense, nutrient-poor foods has led to a rise in “hidden hunger,” where micronutrient deficiencies persist despite adequate or excessive caloric intake [1,9]. In addition, low dietary diversity, high phytate intake, and reduced consumption of nutrient-rich foods further limit mineral availability during the critical growth periods. Lifestyle factors, including reduced physical activity and limited sunlight exposure, may further influence bone accrual and vitamin D status [10,11]. Collectively, these nutritional and lifestyle factors highlight the need for an integrated approach to PBH that considers the adequacy and interaction of multiple nutrients rather than focusing exclusively on individual nutrients.

Although the biological roles of individual nutrients in skeletal development are well established, evidence for the clinical superiority of multi-nutrient supplementation over single-nutrient approaches in children remains limited. An integrated approach may nevertheless be clinically relevant in children with dietary inadequacy or multiple micronutrient deficiencies, given the interdependent roles of nutrients in bone mineralization and skeletal development. Thus, available evidence was combined with multidisciplinary expert input to develop a practical framework for optimizing PBH in the Indian context. The Multi-nutrient Optimization for Skeletal Advancement and Integrated Care in PBH (MOSAIC-PBH) expert consensus was developed using a structured literature review and modified Delphi process, with the aim of providing evidence-informed, clinically applicable recommendations for assessment, prevention, and management of nutritional factors relevant to pediatric skeletal health.

Unmet need

Despite the high burden of micronutrient deficiencies, current approaches to PBH remain largely reductionist, focusing primarily on single-nutrient supplementation, most commonly calcium and vitamin D. While these nutrients are essential, evidence suggests that such strategies have variable and context-dependent effects on bone mineral density (BMD) and fracture outcomes in otherwise healthy children [9,12]. These limitations reflect a gap between clinical practice and the biological complexity of bone metabolism. Bone strength and structure are regulated through coordinated interactions among multiple nutrients and hormonal pathways, rather than isolated nutrient effects [13]. In routine practice, however, assessment and management often remain fragmented, with limited evaluation of coexisting micronutrient deficiencies and inadequate integration of dietary, environmental, and lifestyle factors [9].

This disconnect highlights a critical unmet need for a more comprehensive and physiologically aligned approach to PBH. A framework that recognizes the interdependence of multiple nutrients and integrates nutritional, biochemical, and lifestyle determinants may offer a more comprehensive approach to optimizing bone accrual during growth, particularly in children with dietary inadequacy or multiple micronutrient deficiencies, while acknowledging that direct evidence for the clinical superiority of multi-nutrient strategies over single-nutrient approaches remains limited.

Objectives and scope

The objective of this consensus paper, MOSAIC-PBH, is to provide a comprehensive, evidence-based framework for the assessment, prevention, and management of PBH. This document is intended for pediatricians, neonatologists, endocrinologists, orthopedists, nutritionists, and other healthcare professionals involved in the care of children and adolescents.

Specifically, this expert consensus and framework aims to characterize the burden and key determinants of PBH, with emphasis on high-risk populations and regions with a high prevalence of micronutrient deficiencies. It further seeks to elucidate the role of multi-nutrient interactions and hormonal regulation in skeletal development. In addition, it provides practical guidance on screening, risk stratification, and supplementation strategies for clinical practice, and establishes consensus-based recommendations to support standardized and effective management of PBH.

Methodology

The expert consensus was developed using a structured, modified Delphi methodology integrating a structured literature review, evidence-informed expert discussions, and anonymized voting. An expert advisory board meeting was held on March 1, 2026, in Mumbai, Maharashtra, India, where current evidence on PBH was reviewed through structured presentations and moderated discussions. The process was aligned with established reporting standards, including the ACCORD (ACcurate COnsensus Reporting Document) checklist, to support transparency and methodological rigor [14]. The panel comprised 23 multidisciplinary experts from across India, selected based on their clinical expertise and experience in pediatric care, bone health, endocrinology, orthopedics, nutrition, and growth-related disorders, including pediatricians, neonatologists, pediatric endocrinologists, orthopedic specialists, and pediatric nutrition and growth specialists.

A structured literature review of PubMed/MEDLINE (MEDical Literature Analysis and Retrieval System Online) and other relevant peer-reviewed sources was undertaken to inform the consensus development process. The search covered publications from January 2000 to the most recent available evidence and focused on key domains relevant to PBH, including bone mineralization and accrual, PBM, calcium and vitamin D metabolism, other relevant micronutrients, nutritional inadequacy and deficiency, and environmental and lifestyle determinants. Evidence relevant to the Indian population, including Indian epidemiological and nutritional data, was considered where available. The literature was reviewed and synthesized narratively to inform the expert discussions and development of the consensus statements. The review was intended to provide an evidence base for the expert consensus and was not conducted as a systematic review or meta-analysis.

Following the literature review and expert discussions, 20 statements covering key aspects of PBH and clinical management were finalized for Delphi voting. All 23 panelists participated in the anonymized voting process and independently rated each statement using a five-point Likert scale ranging from strongly agree to strongly disagree. Consensus was predefined as ≥75% agreement, calculated as the proportion of panelists selecting “agree” or “strongly agree.” The distribution of responses across all five Likert categories was recorded for each statement. All 20 statements achieved the predefined consensus threshold in the first round, with agreement ranging from 95% to 100%; therefore, no additional Delphi rounds were required.

## Review

Burden of pediatric bone health and nutritional landscape

PBH is influenced by a complex interplay of nutritional, environmental, and lifestyle factors. A high burden of micronutrient deficiencies, often subclinical, remains a key determinant of impaired skeletal development. Understanding the extent, drivers, and consequences of these deficiencies is essential for effective prevention and management.

Hidden Hunger and Micronutrient Deficiency

Hidden hunger, defined as subclinical micronutrient deficiency despite adequate caloric intake, is a major and often under-recognized contributor to impaired pediatric bone health, particularly in low- and middle-income settings [15]. Deficiencies of key bone-related nutrients, including calcium, vitamin D, zinc, and magnesium, are highly prevalent and are associated with impaired bone mineralization, reduced BMD, and altered skeletal development [9,16]. Importantly, these deficiencies may coexist and may have interrelated effects on skeletal development [13,17]. Despite this, they often remain subclinical and undetected in routine clinical practice, as children may appear anthropometrically normal despite underlying micronutrient inadequacy. This highlights that pediatric bone health may be influenced by the combined nutritional status of multiple micronutrients rather than isolated deficiencies.

Extent of Dietary Inadequacy and Subclinical Deficiency

Dietary inadequacy of key bone-related micronutrients in children exceeds the prevalence of clinically detected biochemical deficiencies, suggesting a substantial burden of subclinical insufficiency. Calcium intake is particularly inadequate, with more than 90% of children estimated to have insufficient intake, while biochemical deficiency is reported in approximately 60% [18-20]. This gap highlights the limitations of relying solely on laboratory markers to assess nutritional status. Intake patterns are also suboptimal, with 70-85% of children and adolescents consuming less than 50% of the recommended dietary allowance (RDA), and only 15-30% achieving more than 70% of the RDA [18,19]. Zinc inadequacy is similarly widespread, affecting approximately 80-85% of children. Reported deficiency varies across age groups, with estimates of around 19% in preschool children and over 30% in adolescents. Regional data from India indicate prevalence rates of 20-47%, influenced by dietary patterns and environmental factors such as soil nutrient content [19,20]. However, data on magnesium status are limited, reflecting the lack of reliable biomarkers and national surveillance. Available evidence suggests that magnesium inadequacy may be common but remains under-characterized [18-20].

Population-level data further support a high underlying nutritional burden. National Family Health Survey (NFHS-5) (2019-21) reports that 35.5% of children under five are stunted, 19.3% are wasted, and 32.1% are underweight [21]. These indicators, although nonspecific, are markers of nutritional and growth-related vulnerability and should not be interpreted as direct measures of micronutrient deficiency. Stunting, in particular, serves as an indicator of long-term nutritional inadequacy and impaired linear growth, with implications for overall growth and potentially bone health. Overall, these findings highlight a gap between dietary intake and detectable deficiency, underscoring the need for broader assessment strategies that account for potential subclinical nutrient insufficiency alongside clinical and biochemical assessment.

Dietary Transitions and Lifestyle Factors

The high burden of micronutrient deficiencies must be understood within the context of ongoing dietary and lifestyle transitions. Rapid urbanization and changing food environments have led to increased consumption of energy-dense, nutrient-poor processed foods and reduced intake of nutrient-rich foods [22]. This shift contributes to inadequate intake of calcium, magnesium, and zinc and may contribute to mineral imbalances, including a higher dietary phosphorus-to-calcium ratio, which may influence bone metabolism through parathyroid hormone (PTH)-mediated pathways [10]. Lifestyle changes further compound these risks. Reduced physical activity and increased sedentary behavior, including prolonged screen time, limit the mechanical loading of bone, which is essential for optimal bone accrual during growth [23]. In addition, limited sunlight exposure due to indoor lifestyles may contribute to inadequate vitamin D status and, consequently, affect calcium absorption and mineral homeostasis [10]. Diets with low mineral density or high phytate content can reduce mineral bioavailability during critical growth periods, thereby potentially affecting skeletal development [16]. Broader determinants, including socioeconomic factors and maternal nutritional status, further influence micronutrient availability and bone health outcomes in children [24]. Collectively, these factors contribute to a complex and evolving nutritional landscape that may adversely affect pediatric bone health.

Clinical Consequences

The clinical consequences of poor nutritional status and micronutrient deficiencies extend across the life course. In early life, these deficiencies can result in impaired bone mineralization, reduced BMD, and clinical conditions with manifestations including skeletal deformities, delayed growth, hypocalcemia, and fractures [24,25]. Chronic undernutrition, reflected as stunting, is associated with reduced bone mineral content, impaired bone mineralization, and compromised skeletal strength [1,16]. Importantly, inadequate nutrition during critical periods of growth may adversely affect PBM acquisition [26]. Reduced bone mass has been associated with increased fracture risk, and dietary patterns such as low milk intake and poor overall diet quality may further contribute to skeletal fragility [22,27]. Early nutritional deficits may have lasting effects on bone structure and strength, with potential implications for skeletal health across the life course [16]. A critical early-life manifestation of deficient mineral accretion is osteopenia of prematurity, predisposing preterm infants to metabolic bone disease and fractures [1,9].

Determinants of pediatric bone health

PBH is influenced not only by nutrient intake but also by environmental, dietary, and lifestyle factors. These factors act together to reduce mineral availability, impair absorption, and disrupt bone metabolism. Their cumulative effect contributes to suboptimal bone mineralization, particularly in children already at risk due to poor nutritional status.

Environmental Determinants of Mineral Availability

Environmental factors such as soil mineral depletion can reduce the nutrient content of food crops, particularly zinc and other essential minerals, thereby contributing to dietary inadequacy in populations dependent on plant-based diets [28]. The widespread use of reverse osmosis (RO) water further reduces the intake of minerals such as calcium and magnesium by removing them during purification [29]. In children with marginal dietary intake, this may contribute to cumulative mineral deficiencies and affect calcium homeostasis and bone metabolism [29,30].

Dietary Factors Influencing Mineral Bioavailability

Mineral bioavailability is a key determinant of effective nutrient status. Diets high in phytates, commonly seen in cereal-based patterns, reduce the absorption of calcium, zinc, and iron [31,32]. As a result, even adequate intake may not translate into sufficient mineral availability for bone development. This is particularly important during early life. Complementary feeding practices often provide low mineral density and high phytate content, limiting both intake and absorption [16,33,34]. Preterm infants are especially vulnerable, as inadequate postnatal mineral supply may increase the risk of osteopenia of prematurity [9]. Additional factors, such as lactose intolerance, can reduce dairy intake and calcium availability, while conditions like celiac disease impair the absorption of multiple bone-relevant nutrients, leading to reduced BMD if untreated [26,35].

Lifestyle Determinants

Lifestyle factors significantly influence bone health through their effects on vitamin D status and mechanical loading. Reduced sunlight exposure due to indoor lifestyles contributes to widespread vitamin D deficiency, impairing calcium absorption and bone mineralization [10,36]. Physical activity, particularly weight-bearing exercise, is essential for bone accrual. Sedentary behavior and increased screen time reduce mechanical stimulation of bone and are often associated with lower vitamin D levels, further affecting bone mineralization [10,37].

Physiology and life-course bone development

PBH is governed by dynamic biological processes that regulate skeletal growth, structure, and strength across the life course. Understanding these processes is essential to identifying critical periods and determinants of optimal bone mass accrual.

Bone Modeling and Remodeling

Bone is a dynamic, metabolically active tissue that undergoes continuous structural adaptation through bone modeling and remodeling, processes fundamental to skeletal growth and maintenance. During childhood and adolescence, bone modeling predominates and is responsible for increases in bone size and shape, as well as structural adaptation to mechanical forces [38,39]. In contrast, bone remodeling is a lifelong process in which bone resorption and formation are tightly coupled within remodeling units. This process serves to maintain skeletal integrity, repair microdamage, and regulate mineral homeostasis [40,41]. These processes are highly active during growth and are influenced by nutritional status, hormonal signals, and mechanical loading. Disruptions in these processes, due to nutrient deficiencies or hormonal imbalance, can impair bone accrual and compromise skeletal strength [38].

Bone Accrual Across Early Life Stages

Bone development follows a life-course trajectory, with specific periods of accelerated growth. Adequate intake and absorption of calcium and phosphorus are essential for supporting this rapid bone mineralization [41].

Infancy: A key determinant of bone health in early infancy is intrauterine mineral accretion. The majority of fetal calcium and phosphorus transfer occurs during the third trimester, and preterm birth disrupts this process, resulting in reduced mineral stores and increased risk of osteopenia of prematurity, characterized by impaired bone mineralization, increased fracture risk, and long-term skeletal deficits [9,38].

Childhood: During childhood, bone accrual continues steadily, driven by growth in bone size and gradual increases in BMD. Mechanical loading through physical activity and adequate nutritional intake are critical determinants of bone strength during this stage [38]. Hormonal influences, including growth hormone (GH) and insulin-like growth factor-1 (IGF-1), play a central role in stimulating bone formation and longitudinal growth, while nutritional adequacy ensures the availability of substrates required for mineralization [38].

Adolescence: Adolescence is characterized by accelerated bone accrual, accounting for a substantial proportion of PBM acquisition. Approximately 40-60% of adult bone mass is accrued during adolescence, and over 80% of PBM is achieved by late adolescence, highlighting early life as a critical window for skeletal development [3,42]. This phase is closely associated with the pubertal growth spurt, characterized by rapid growth, increased calcium requirements, and hormonal changes, including increased bone turnover and endocrine activity such as elevated PTH and active vitamin D metabolites, all of which contribute to accelerated skeletal development [43]. Inadequate nutrition during this critical window may result in suboptimal peak bone mass that cannot be fully compensated later in life [44].

Bone mineral accrual follows a life-course trajectory that extends beyond early growth phases into adulthood, with distinct stages characterized by varying rates of bone formation and remodeling. Understanding these stage-specific patterns is essential for identifying critical windows for intervention and optimizing peak bone mass. The patterns of bone accrual and their clinical implications across life stages are summarized in Table 1.

**Table 1:** Bone mineral accrual across early life stages and clinical implications References: [9,38,41,42,43,44] PBM: peak bone mass

Life Stage	Bone Mineral Accrual Pattern	Clinical Implications
Infancy (0-2 years)	Rapid bone growth driven by early linear growth and active bone modeling	Adequate nutrition (calcium, phosphorus, protein, and micronutrients) is critical for establishing the foundation of lifelong skeletal strength
Childhood (3-9 years)	Steady bone accrual at a slower rate compared with puberty	Sustained adequate intake of micronutrients supports continued bone development and contributes to PBM
Adolescence (~10-18 years)	Accelerated bone mineral accrual; major window for PBM acquisition (approximately 40-60% of adult bone mass)	Nutritional adequacy (particularly calcium, vitamin D, and protein) and timely puberty are critical; deficiencies during this phase may have long-term consequences

Determinants and Mechanistic Drivers of Peak Bone Mass Accrual

Genetic and environmental factors: PBM is determined by a combination of genetic and environmental factors. Genetic influences account for a substantial proportion of the variability in PBM, affecting bone size, density, and structure, while environmental factors such as nutrition, physical activity, and lifestyle modulate these genetic predispositions, accounting for ~20-40 % of the variance in PBM [38,39,41]. Nutritional factors play a central role in PBM acquisition, with adequate intake of calcium, phosphorus, protein, and micronutrients being essential for optimal bone mineralization. Deficiencies of these nutrients have been associated with impaired bone accrual and reduced PBM [41].

Hormonal axis: Hormonal regulators, including growth hormone, sex steroids, vitamin D, and PTH, interact to control bone growth, mineral homeostasis, and skeletal maturation, highlighting the integrated nature of PBM determination [38]. GH stimulates bone formation and growth through IGF-1, while vitamin D enhances intestinal calcium absorption and maintains mineral homeostasis [38]. PTH plays a dual role by regulating calcium levels and stimulating bone remodeling. During periods of rapid growth, increased PTH and active vitamin D levels are associated with higher bone accrual rates, reflecting adaptive responses to increased calcium demands [43].

Nutrient availability and interactions: Adequate availability of calcium and phosphorus is essential for hydroxyapatite formation and bone mineralization, while magnesium and zinc modulate enzymatic processes and osteoblast function. Deficiencies in these nutrients can disrupt bone formation and reduce PBM [41]. Importantly, nutrient interactions play a critical role, as imbalances in calcium-phosphorus ratios or deficiencies in regulatory minerals can impair hormonal signaling and bone metabolism.

Impaired calcium-phosphorus accretion in preterm infants: In preterm infants, disrupted intrauterine mineral accretion leads to inadequate calcium and phosphorus stores, resulting in impaired bone mineralization and altered hormonal regulation. This condition contributes to osteopenia of prematurity, in which insufficient mineral availability and a dysregulated PTH-vitamin D axis further compromise skeletal development [38].

Nutrient biology of pediatric bone health

Bone health in children is regulated by a complex interplay of nutrients and hormonal pathways that together support skeletal growth, mineralization, and structural integrity. Rather than acting in isolation, nutrients function within an integrated biological network in which hormonal regulators, structural minerals, and regulatory micronutrients interact to maintain bone metabolism [45,46]. This systems-based perspective provides a more accurate representation of bone physiology compared with traditional single-nutrient models.

Vitamin D as a Central Hormonal Regulator

Vitamin D plays a central role in pediatric bone health and is best understood as a secosteroid hormone with endocrine, paracrine, and autocrine functions [47,48]. Following cutaneous synthesis or dietary intake, vitamin D undergoes hepatic and renal hydroxylation to form its active metabolite, 1,25-dihydroxyvitamin D (calcitriol), which binds to the vitamin D receptor (VDR) to regulate gene expression across multiple tissues [47]. The widespread distribution of VDR underscores its systemic role in mineral metabolism and skeletal development.

A primary function of vitamin D is the regulation of calcium and phosphorus homeostasis. It enhances intestinal absorption of calcium and phosphate and contributes to renal handling of these minerals, ensuring adequate substrate availability for bone mineralization [47,49]. In states of deficiency, reduced calcium absorption leads to hypocalcemia, triggering compensatory increases in PTH [50,51]. This secondary hyperparathyroidism increases bone resorption to maintain serum calcium levels, thereby impairing skeletal mineralization. Vitamin D also interacts with fibroblast growth factor 23 (FGF23) and renal phosphate regulation, further highlighting its role in maintaining mineral balance [52]. Vitamin D functions within a broader endocrine network involving PTH and the GH-IGF-1 axis. Reduced vitamin D levels are associated with elevated PTH, while adequate vitamin D suppresses excessive PTH secretion and supports balanced bone remodeling [51,53]. In addition, vitamin D may influence growth plate activity and longitudinal bone growth through interactions with the GH-IGF-1 axis [47].

Clinically, vitamin D deficiency is associated with impaired mineralization, leading to rickets, delayed growth, skeletal deformities, hypocalcemia, and an increased risk of fractures [47,54]. Biochemically, vitamin D deficiency is characterized by hypocalcemia or hypophosphatemia, elevated alkaline phosphatase, and increased PTH levels [50]. Despite its central role, the biological effectiveness of vitamin D depends on adequate availability of substrate minerals and cofactors, emphasizing that it operates within an integrated nutrient system rather than as an isolated factor [55].

Structural and Regulatory Minerals

Optimal bone mineralization depends on the coordinated availability of key minerals that contribute to both structural integrity and metabolic regulation. Calcium and phosphorus are the principal structural components of bone, forming hydroxyapatite crystals that provide strength and rigidity to the skeletal matrix [55,56]. Approximately 99% of body calcium and 85% of phosphorus are stored in bone, highlighting their essential role in skeletal development. Effective mineralization requires not only adequate intake but also appropriate availability and balance of calcium and phosphorus [56]. Imbalances, particularly low calcium intake in the context of high phosphorus consumption, may influence PTH secretion, bone resorption, and skeletal mineralization [55].

Magnesium is a critical regulatory mineral involved in both bone structure and metabolic processes. It contributes to hydroxyapatite formation and influences bone quality and strength [57]. In addition, magnesium acts as a cofactor in vitamin D activation and plays a role in PTH regulation, thereby contributing to calcium homeostasis [55]. Deficiency may disrupt these processes, leading to impaired mineral balance and suboptimal bone mineralization. Clinical evidence indicates a positive association between magnesium status and BMD in children, although it remains under-recognized in routine clinical assessment [58].

Zinc is an essential trace element that supports bone formation and growth by influencing osteoblast proliferation, differentiation, and activity. It is also involved in collagen synthesis and bone matrix production. Zinc inhibits osteoclast-mediated bone resorption, thereby promoting a net anabolic effect on bone [59]. Deficiency has been associated with impaired linear growth, delayed skeletal development, and reduced bone mass in children [58].

Together, these minerals perform complementary roles, with structural minerals providing the physical matrix of bone and regulatory minerals supporting enzymatic activity, cellular function, and hormonal interactions necessary for skeletal development.

Nutrient Interactions and Functional Integration

Bone metabolism is governed by dynamic interactions among nutrients and endocrine regulators. The vitamin D-calcium-phosphorus axis represents the central pathway for bone mineralization, with vitamin D facilitating intestinal absorption of calcium and phosphate, while PTH and FGF23 regulate their systemic balance [55]. Beyond this central axis, regulatory minerals modulate key metabolic pathways. Magnesium is required for the activation of vitamin D, and its deficiency may reduce vitamin D effectiveness and impair calcium absorption [55]. Zinc contributes to osteoblast function and bone matrix synthesis, linking micronutrient status to skeletal growth [55,60]. Nutrient interactions also include competitive and potentially synergistic effects; such as, excessive calcium intake may interfere with zinc absorption, highlighting the importance of balanced intake [61]. These interactions illustrate the physiological interdependence of nutrients, although the extent to which such interactions translate into clinically meaningful additive or synergistic effects of multi-nutrient supplementation in children remains uncertain [61].

Systems-Based Model of Bone Health

A systems-based model emphasizes that optimal pediatric bone health depends on the balanced and integrated availability of multiple nutrients [45]. Within this framework, nutrients function collectively as part of an interconnected network rather than independent factors. Micronutrient deficiencies often coexist and may lead to a cascade of physiological disturbances, in which disruption of one pathway can result in secondary disturbances in other pathways. For example, vitamin D deficiency reduces calcium absorption, leading to secondary hyperparathyroidism and increased bone resorption. Similarly, magnesium deficiency can impair vitamin D activation, further compounding calcium imbalance. Coexisting deficiencies of calcium, vitamin D, zinc, and magnesium may occur in pediatric populations and could have cumulative effects on bone accrual and peak bone mass, although direct evidence demonstrating synergistic clinical effects remains limited [55]. This integrated framework is particularly relevant in early-life conditions such as osteopenia of prematurity, where disrupted mineral accretion, immature metabolic pathways, and inadequate nutrient supply converge to impair bone mineralization [62,63]. Overall, these observations support consideration of a comprehensive, multi-nutrient framework alongside assessment of individual nutrient status, reflecting the physiological complexity of bone metabolism and providing a conceptual foundation for clinical management.

Maternal-Fetal Mineral Axis and Early-Life Programming

The role of mineral nutrition in PBH extends beyond postnatal life and is established as early as the preconception and intrauterine periods. The “first 1000 days,” spanning from conception to early childhood, represents a critical window during which nutritional exposures influence fetal growth, skeletal development, and long-term health outcomes [64,65]. Disruptions in maternal mineral status, particularly involving calcium, magnesium, and zinc, may adversely affect placental function, fetal mineral accretion, and early metabolic programming [64,65].

Emerging evidence indicates that inadequate maternal micronutrient status is associated with adverse pregnancy outcomes, including preterm birth, intrauterine growth restriction, and impaired neurodevelopment, as well as suboptimal postnatal growth and increased susceptibility to infections and metabolic dysfunction [64,65]. These effects are mediated through alterations in cellular proliferation, enzymatic activity, and endocrine regulation, and are consistent with the developmental origins of health and disease (DOHaD) paradigm [64,65]. The influence of mineral nutrition extends beyond birth, with adequate intake during infancy and early childhood remaining essential to support rapid growth, skeletal mineralization, neurodevelopment, and immune function. Together, these observations highlight the importance of a life-course approach to bone health that integrates maternal nutrition with early childhood nutritional strategies [1,60]. The stage-specific influence of key minerals on pregnancy outcomes and child growth and development is summarized in Table 2.

**Table 2:** Impact of deficiencies of key minerals on pregnancy outcomes and child growth and development References: [64,65]

Mineral	Impact on Pregnancy and Fetal Development	Impact on Child Growth and Development
Zinc	Increased risk of preterm birth Adverse birth outcomes and delivery complications Impaired neurodevelopment	Impaired epithelial barrier function (skin and mucosa) Increased susceptibility and severity of infections Growth impairment Altered immune and physiological responses
Calcium	Increased risk of pre-eclampsia Preterm birth Intrauterine growth restriction Low birth weight	Nutritional rickets Reduced bone mineralization Dental enamel hypoplasia Behavioral and neurodevelopmental disturbances
Magnesium	Increased risk of severe pre-eclampsia Preterm birth Low Apgar scores Hypoxic-ischemic complications	Hypocalcemia Impaired bone growth Reduced cognitive function and processing ability Poor attention and concentration

Clinical approach to pediatric bone health

A comprehensive clinical approach to PBH requires integration of risk-based screening, multidimensional assessment, and targeted preventive and therapeutic strategies. Given the multifactorial nature of bone development and the potential for coexisting micronutrient inadequacies, management should consider factors beyond isolated nutrient correction within a holistic, individualized framework.

Risk-Based Screening and Identification of At-Risk Populations

Routine universal screening for bone health or micronutrient deficiencies is not recommended in otherwise healthy children due to limited evidence of benefit and concerns regarding cost-effectiveness [9,66]. Instead, a targeted, risk-based approach is recommended, focusing on children with identifiable clinical, nutritional, or environmental risk factors for impaired bone mineralization [9]. Major at-risk groups include children with chronic illnesses such as inflammatory disorders, endocrine conditions, malabsorption syndromes, and chronic kidney disease, which may affect nutrient absorption, hormonal regulation, or bone turnover. Children receiving long-term medications, including glucocorticoids, antiepileptics, or proton pump inhibitors, are also at increased risk of reduced bone mass [67]. Nutritional risk factors, such as low dietary calcium intake, vitamin D deficiency, poor dietary diversity, and conditions associated with micronutrient inadequacy, are particularly relevant in populations with a high burden of nutritional inadequacy and hidden hunger [9,68]. Clinical indicators, including recurrent or low-energy fractures, delayed growth, and features suggestive of rickets or osteomalacia, warrant further evaluation [67,69]. Preterm and very low birth weight infants represent a particularly vulnerable group due to disrupted intrauterine mineral accretion, resulting in reduced calcium and phosphorus stores and increased susceptibility to osteopenia of prematurity [67]. Identification of at-risk children should therefore be based on a comprehensive evaluation of clinical history, dietary intake, environmental exposures, and early-life factors. Key at-risk groups and clinical indicators for targeted screening are summarized in Table 3 to facilitate clinical application.

**Table 3:** Risk-based identification of children at risk for impaired bone health References: [9,66,67,68,69] CKD: chronic kidney disease

Risk Category	At-Risk Population / Factors	Clinical Relevance
Chronic medical conditions	Inflammatory disorders, endocrine diseases, malabsorption syndromes, CKD	Impaired nutrient absorption, altered hormonal regulation, and disrupted bone turnover
Long-term medication use	Glucocorticoids, antiepileptics, proton pump inhibitors	Reduced bone mineralization and increased risk of low bone mass
Nutritional risk factors	Low dietary calcium intake, vitamin D deficiency, poor dietary diversity, micronutrient inadequacy	Inadequate substrate and cofactors for bone mineralization
Clinical indicators	Recurrent or low-energy fractures, delayed growth, features of rickets or osteomalacia	May suggest underlying skeletal fragility or metabolic bone disease
Early-life factors	Preterm birth, very low birth weight	Reduced intrauterine mineral accretion and increased risk of osteopenia of prematurity
Environmental and lifestyle factors	Limited sunlight exposure, low physical activity, poor diet quality	MAy contribute to vitamin D deficiency and suboptimal bone accrual

Clinical and Biochemical Assessment

Assessment of PBH requires a combination of clinical evaluation and targeted investigations, as no single parameter adequately reflects bone status [67,70]. Clinical evaluation should include growth patterns, dietary intake, physical activity, sunlight exposure, and fracture history, which together provide essential context for identifying underlying risk factors [67,70]. Physical examination may reveal signs of skeletal compromise, including limb deformities, delayed growth, or features suggestive of rickets [67,70]. Biochemical assessment provides insight into mineral metabolism and should include serum 25-hydroxyvitamin D, calcium, phosphorus, magnesium, alkaline phosphatase, and PTH, as indicated [71]. These markers help identify disturbances in the vitamin D-calcium-phosphorus axis but must be interpreted in conjunction with clinical findings, as they do not directly reflect bone strength or fracture risk. Importantly, evaluation should consider the broader nutritional context rather than relying solely on isolated biomarkers, particularly when dietary inadequacy or risk factors for multiple micronutrient deficiencies are present. BMD assessment using dual-energy X-ray absorptiometry (DXA) may be considered in selected cases [9]. However, interpretation in children requires adjustment for age, sex, and pubertal status, and results should not be used in isolation for diagnosis, given that peak bone mass has not yet been achieved [72].

Preventive Strategies and Nutritional Optimization

Preventive strategies should adopt a life-course approach, ensuring adequate nutrient availability during critical periods of growth and bone accrual. Interventions should integrate dietary optimization, appropriate supplementation, and lifestyle modification. Adequate intake of calcium and vitamin D remains fundamental for maintaining bone health and preventing rickets, particularly in children with limited dietary intake or inadequate sunlight exposure [73-75]. Preventive supplementation is especially relevant in high-risk groups, including exclusively breastfed infants, children with poor dietary intake, and those with limited sun exposure [74,76]. However, preventive strategies should also consider overall dietary adequacy and the potential contribution of other micronutrients. Ensuring adequate intake of multiple micronutrients, including magnesium and zinc, is biologically relevant to bone metabolism and growth, although evidence for specific multi-nutrient supplementation strategies in otherwise healthy children remains limited [60]. Lifestyle factors are equally important, as regular weight-bearing physical activity and adequate sunlight exposure play key roles in promoting bone accrual and maintaining vitamin D status [37].

Therapeutic Supplementation and Management

Therapeutic interventions are indicated in children with documented deficiencies, clinical manifestations such as rickets, or conditions associated with impaired bone health. Correction of vitamin D deficiency through supplementation is effective in restoring calcium absorption and reversing biochemical abnormalities associated with impaired mineralization [76]. Calcium supplementation, particularly when combined with vitamin D, has been shown to increase bone mineral content and support skeletal recovery in populations with deficiencies [74]. However, therapeutic strategies should consider the possibility of coexisting micronutrient deficiencies, as correction of a single nutrient may be insufficient when broader nutritional inadequacies are present [77]. Available evidence on combined supplementation approaches remains limited and heterogeneous, and superiority over single-nutrient strategies has not been consistently established. Although addition of zinc to calcium and vitamin D supplementation has been associated with greater bone mineral accrual in some pediatric populations, these findings should not be interpreted as definitive evidence of synergistic clinical effects [73]. Therapeutic supplementation should therefore be individualized, guided by clinical assessment, biochemical findings, and underlying risk factors, with emphasis on correcting both primary and coexisting deficiencies.

Variability in Diagnostic Thresholds and Therapeutic Approaches for Vitamin D

There is no universal consensus on the definition of vitamin D deficiency, with substantial variability across guidelines based on serum 25-hydroxyvitamin D (25(OH)D) thresholds. Major bodies such as the Institute of Medicine (IOM) and American Academy of Pediatrics (AAP) define deficiency at levels below 20 ng/mL, whereas the Endocrine Society and related organizations recommend higher thresholds, often considering levels below 30 ng/mL as insufficient and targeting ≥30 ng/mL for sufficiency [54,78,79]. Very low levels of vitamin D (<10-12 ng/mL) are consistently associated with increased risk of rickets and osteomalacia; however, classification of intermediate levels remains inconsistent [79,80]. This lack of standardization contributes to variability in diagnosis and may result in both under-recognition and overdiagnosis in clinical practice.

Differences in diagnostic thresholds translate directly into variability in therapeutic targets and supplementation strategies. Preventive dosing generally ranges from 400 IU/day in infancy to 600-1000 IU/day in older children and adolescents, depending on age and risk profile [66,81]. In contrast, treatment regimens for deficiency vary widely, including daily high-dose therapy (e.g., 2000-6000 IU/day) or intermittent high-dose regimens such as weekly bolus dosing (60,000 IU once weekly) [79,82]. Recommendations regarding upper intake limits and safety thresholds also differ across organizations, further contributing to inconsistency in prescribing practices [83].

Given these variations, vitamin D management in pediatric populations should be individualized and context-specific. Clinical decision-making should consider age, baseline nutritional status, dietary calcium intake, sunlight exposure, comorbid conditions, and regional prevalence of deficiency. Importantly, vitamin D should not be addressed in isolation, as its biological effectiveness depends on adequate availability of calcium and other micronutrients. Accordingly, vitamin D supplementation should be considered within the broader nutritional context, including adequacy of calcium and other relevant micronutrients, when managing children at risk of or with documented deficiency [84].

Variability in Diagnostic Thresholds and Therapeutic Approaches for Other Key Minerals

In contrast to vitamin D, standardized diagnostic thresholds for other key bone-related micronutrients, including calcium, zinc, and magnesium, are less well defined in pediatric practice. Serum calcium is tightly regulated within a narrow physiological range (approximately 8.5-10.5 mg/dL), and hypocalcemia is typically defined only when levels fall below ~8.5 mg/dL, often occurring late in the course of deficiency [55,85]. As a result, serum calcium frequently remains within the normal range despite significant dietary inadequacy, limiting its utility as a marker of nutritional status [55]. Similarly, zinc deficiency is defined using variable serum cut-offs, commonly <70 µg/dL in younger children and approximately 70-74 µg/dL in older children and adolescents, although these thresholds vary across studies and are influenced by factors such as age, fasting status, inflammation, and diurnal variation [86,87]. Magnesium assessment is further complicated by wide reference ranges and poor sensitivity of serum levels. Typical serum reference ranges are approximately 1.7-2.2 mg/dL; however, only ~1% of total body magnesium is present in serum, making it an insensitive marker of total body stores [88,89].

These limitations contribute to variability in clinical interpretation and management. Unlike vitamin D, where diagnostic thresholds and treatment regimens are relatively well established, recommendations for calcium, zinc, and magnesium are largely based on dietary reference intakes rather than clearly defined therapeutic thresholds [90]. The absence of standardized diagnostic criteria, combined with limited pediatric interventional data, results in heterogeneous clinical practices, with supplementation often guided by clinical judgment rather than evidence-based protocols.

Life-Stage and Risk-Stratified Approach

Nutritional requirements for bone health vary across developmental stages, necessitating age-specific and risk-based strategies. In infancy, rapid growth and dependence on dietary intake make supplementation particularly important. Vitamin D supplementation is widely recommended to prevent deficiency and support bone mineralization [74]. Preterm infants require targeted supplementation, including calcium and phosphorus, to address deficits resulting from interrupted intrauterine accretion [91]. During childhood, steady bone accrual requires sustained intake of essential nutrients along with adequate physical activity. Preventive strategies should focus on maintaining dietary adequacy and addressing emerging deficiencies [60,74]. Adolescence represents a critical window for bone mass accrual, with increased nutritional requirements. Adequate intake of calcium, vitamin D, and other micronutrients during this stage is important for supporting peak bone mass accrual and long-term skeletal health [74].

To support clinical decision-making, age-stratified safety anchors for therapeutic supplementation of key micronutrients to optimize pediatric bone health are summarized in Table 4. These values are derived from the Indian Council of Medical Research - National Institute of Nutrition (ICMR-NIN) 2020 (Updated 2024) Tolerable Upper Limits (TUL) and represent safe intake ranges, not recommended therapeutic dosing, and should be interpreted in the context of individual clinical status and nutritional assessment [92].

**Table 4:** Age-stratified tolerable upper intake limits (TULs) for selected micronutrients in children and adolescents *For magnesium, TUL applies only to supplemental (non-dietary) intake. Information Source: ICMR-NIN Expert Group on Nutrient Requirement for Indians, 2020 (updated 2024) [92]

Age Group	Calcium TUL (mg/day)	Magnesium TUL (mg/day)*	Zinc TUL (mg/day)	Vitamin D TUL (IU/day)
0-6 months	—	—	4	1000
6-12 months	—	—	5	1500
1-3 years	1500	65	7	2500
4-6 years	2500	110	12	3000
7-9 years	2500	110	12	3000
10-12 years	3000	350	23	4000
13-15 years	3000	350	34	4000
16-18 years	3000	350	34	4000

Integrated Multi-Nutrient Supplementation Strategy

Given the potential for coexisting micronutrient inadequacies, a multi-nutrient and multi-mineral framework may provide a physiologically informed approach to PBH, particularly when multiple nutritional risks are present [60]. The biological rationale for combined supplementation lies in the interdependent roles of nutrients, where calcium and phosphorus provide structural support, vitamin D regulates their absorption and homeostasis, while magnesium and zinc contribute to enzymatic activity and cellular processes essential for bone formation [45,60]. Addressing documented or suspected coexisting deficiencies may be important, as imbalances in one nutrient may influence the physiological effects of others. Magnesium deficiency may impair vitamin D activation, while zinc deficiency may affect osteoblast activity, providing a biological rationale for considering these nutrients when broader nutritional inadequacy is present [55]. Such integrated approaches are particularly relevant in high-risk populations, including children with poor dietary diversity and preterm infants, where multiple nutritional deficits may coexist and contribute to impaired bone mineralization [62,63].

Population-Level and Context-Specific Strategies

Supplementation strategies may be implemented at both individual and population levels, depending on the burden of deficiency and available resources. Population-level interventions, including food fortification and broad supplementation programs, may be appropriate in settings with widespread micronutrient deficiencies [61]. In contrast, risk-based approaches allow targeted intervention in high-risk groups, improving efficiency and minimizing unnecessary supplementation. A combined strategy integrating population-level measures with targeted clinical interventions may be appropriate, particularly in regions with heterogeneous risk profiles. The effectiveness of supplementation is closely linked to underlying dietary patterns. Diets low in nutrient density and high in refined carbohydrates are associated with poor micronutrient intake and adverse bone health outcomes [22]. Supplementation should therefore complement, rather than replace, efforts to improve dietary quality and diversity [61].

Limitations of Current Clinical Approaches

Despite advances in understanding PBH, several limitations persist in current clinical practice. Screening strategies often rely on isolated biomarkers, particularly vitamin D levels, without adequately considering coexisting deficiencies and nutrient interactions. This reductionist approach may fail to capture the complexity of bone metabolism [66]. In addition, routine micronutrient screening is rarely performed due to cost constraints and the lack of standardized, clinically validated cut-offs, further limiting comprehensive assessment of nutritional status [9,66]. Biochemical markers provide indirect measures and may not correlate directly with bone strength or fracture risk. Similarly, DXA interpretation in children is complicated by growth-related changes and may lead to misclassification [72,93]. In addition, many children with micronutrient deficiencies remain asymptomatic, delaying diagnosis and intervention. These limitations support consideration of integrated, context-specific, and risk-based clinical frameworks that incorporate nutritional, environmental, and developmental factors.

Implementation considerations for multi-mineral framework in pediatric bone health

Effective implementation of multi-nutrient and multi-mineral strategies in PBH requires careful consideration of formulation design, dosing, and clinical usability. The success of supplementation depends not only on nutrient composition but also on bioavailability, tolerability, and adherence, all of which can influence its use and effectiveness in clinical practice.

Formulation Design and Bioavailability

Bioavailability is a critical determinant of clinical effectiveness and refers to the proportion of an ingested nutrient that is absorbed and utilized by the body. It is influenced by multiple factors, including chemical form (salt), solubility, dietary matrix, and interactions with other nutrients [94]. Different mineral salts exhibit variable absorption profiles. Organic salts of magnesium (e.g., citrate, lactate, aspartate) exhibit higher bioavailability than inorganic forms, such as magnesium oxide, due to greater solubility [95]. Similarly, zinc formulations such as zinc gluconate and zinc glycinate are better absorbed than less soluble forms like zinc oxide [96].

The dietary matrix further modifies absorption; phytates reduce mineral bioavailability, whereas certain proteins and organic acids may enhance it [97,98]. In addition to absorption, tolerability varies across formulations; poorly absorbed salts may cause gastrointestinal side effects such as diarrhea, whereas more bioavailable forms are generally considered to have favorable absorption and tolerability profiles [95].

In the context of multi-nutrient and multimineral supplementation, formulation design should account for nutrient interactions. Appropriate formulation design may help optimize nutrient delivery and minimize clinically relevant competition for absorption, whereas poorly designed formulations may result in competitive inhibition and reduced absorption [55,95,99].

Dosing Strategies and Nutrient Interactions

The effectiveness of supplementation depends on appropriate elemental dosing rather than the total weight of the compound. For example, magnesium content varies substantially across salt forms, and dosing must be based on elemental magnesium to ensure adequacy [95]. Dosing strategies should also consider age-specific requirements, physiological demands during growth, and tolerable upper intake levels, as excessive intake may lead to adverse effects, including gastrointestinal intolerance or mineral imbalances [95]. Nutrient interactions are particularly relevant in multi-mineral supplementation. High doses of one mineral may interfere with the absorption of another, necessitating balanced dosing and consideration of the relevant nutrient interactions [55,99]. A rational dosing approach, therefore, requires alignment with physiological requirements while minimizing antagonistic interactions and avoiding assumptions of synergistic clinical effects unless supported by evidence.

Clinical Usability, Adherence, and Prescribing Considerations

The practical effectiveness of supplementation is influenced by adherence, particularly in pediatric populations where long-term supplementation may be required. Palatability, dosage form, and gastrointestinal tolerability are key determinants of compliance [60]. Child-friendly formulations, including syrups, dispersible tablets, and chewable preparations, are generally associated with better acceptance [100,101]. Implementation in clinical practice should be individualized and guided by risk assessment, dietary intake, and biochemical findings rather than a uniform approach. Factors such as age, developmental stage, comorbidities, and likelihood of multiple micronutrient deficiencies should inform the choice of formulation and supplementation strategy [102]. The timing of administration may influence absorption, as interactions with food components or medications can affect bioavailability. Certain mineral supplements may need to be spaced from specific drugs to avoid reduced absorption [94]. Regular monitoring is important, particularly in therapeutic settings, to assess response and adjust dosing as needed. Simplified regimens and appropriately designed combination formulations may improve adherence, whereas overly complex or poorly designed regimens may reduce compliance and effectiveness. Overall, implementation strategies should align with the multi-nutrient framework, ensuring coordinated delivery of nutrients in forms that are bioavailable, well-tolerated, and acceptable to children, while recognizing that formulation characteristics alone do not establish superior clinical outcomes.

Evidence gaps and research priorities

Despite advances in understanding pediatric bone health, significant gaps remain across clinical, epidemiological, and translational domains. Most existing research has focused on single-nutrient interventions, particularly vitamin D and calcium, with limited high-quality randomized controlled trials evaluating multi-nutrient and multi-mineral strategies, despite a strong biological rationale for interactions among nutrients and the potential for complementary effects [66,103,104]. There is also a lack of robust, region-specific epidemiological data, especially from India, on the prevalence and co-occurrence of deficiencies beyond vitamin D, including calcium, magnesium, and zinc, as well as their associations with bone outcomes and peak bone mass [105]. Evidence for the role of regulatory minerals such as magnesium and zinc is largely derived from observational or adult studies, with insufficient pediatric interventional data to establish clear clinical recommendations [58,106]. Additionally, although the importance of calcium-phosphorus balance is well recognized, there is limited clinical validation of optimal calcium:phosphorus ratios across diverse dietary contexts [48]. Environmental determinants, including soil micronutrient depletion, water mineral content, phytate-rich diets, and lifestyle factors, are increasingly acknowledged but remain underexplored in integrated research frameworks [23]. Collectively, these gaps highlight the need for well-designed multi-nutrient clinical trials, comprehensive, context-specific epidemiological studies, deeper investigation of regulatory minerals and nutrient interactions, integration of environmental determinants into research models, and development of standardized, evidence-informed policies to guide pediatric bone health management.

Delphi outcomes and consensus statements

All 20 statements achieved the predefined consensus threshold (≥75% agreement) in the first round of voting, and no statements required revision or additional Delphi rounds. Agreement across statements ranged from 95% to 100%, based on the proportion of panelists selecting “agree” or “strongly agree.” Responses are analyzed descriptively, and agreement levels are reported as percentages across Likert scale categories. The observed agreement indicates substantial alignment among the participating experts on key aspects of pediatric bone health, including the importance of early-life nutrition, the role of multi-nutrient interactions, and the need for integrated management approaches. These finalized statements form the basis of the recommendations presented (Table 5).

**Table 5:** Consensus statements and Delphi polling outcomes *The level of agreement combines strongly agree and agree votes percentage

No.	Consensus Statements	Level of Agreement*
Foundational		
1	Achieving optimal bone health during childhood and adolescence is important for maximizing lifetime peak bone mass and reducing fracture risk in adulthood. Strongly agree: 91%, Agree: 9%	100%
2	Normal bone mineralization in children during growth requires adequate and co- availability of calcium, phosphorus, magnesium, zinc, and vitamin D for structural formation, mineral deposition, and hormonal regulation. Strongly agree: 100%	100%
3	Balanced ratios of minerals and sufficient cofactor availability are required for optimal bone accretion, and deficiency or imbalance of even a single key nutrient may adversely affect skeletal outcomes. Strongly agree: 74%, Agree: 22%, Neither agree nor disagree: 4%	96%
Hidden Hunger and Multi-Mineral Deficiency		
4	Hidden hunger, characterized by subclinical deficiencies of multiple micronutrients, is common in children and may impair skeletal development, even in those with normal growth parameters. Strongly agree: 65%, Agree: 35%	100%
5	Diets characterized by low diversity and high intake of nutrient-poor processed foods increase the risk of concurrent mineral insufficiencies relevant to bone health. Strongly agree: 83%, Agree: 17%	100%
6	A focused dietary assessment should be part of routine pediatric evaluation to identify children at risk of mineral inadequacy. Strongly agree: 74%, Agree: 26%	100%
Calcium and Other Minerals		
7	Calcium is fundamental for bone matrix mineralization; however, optimal skeletal development depends on concurrent adequacy of complementary minerals and cofactors. Strongly agree: 87%, Agree: 13%	100%
8	In children with existing or suspected multiple micronutrient deficiencies, multi-mineral supplementation may more appropriately address skeletal requirements than single-nutrient supplementation. Strongly agree: 65%, Agree: 30%, Disagree: 4%	96%
Life-Stage Windows for Intervention		
9	Infancy and early childhood represent critical periods of mineral accretion during which nutritional deficits may have long-term consequences for peak bone mass. Strongly agree: 57%, Agree: 39%, Neither agree nor disagree: 4%	96%
10	Adolescence represents a priority window for bone health intervention, as a large proportion of adult bone mass is accrued during this stage. Strongly agree: 83%, Agree: 17%	100%
11	Preventive and therapeutic supplementation strategies should consider age-specific mineral requirements across developmental stages. Strongly agree: 74%, Agree: 26%	100%
Screening and Risk-Based Strategies		
12	Laboratory evaluation for mineral status should be considered in children with persistent dietary inadequacy, rapid growth, limited sun exposure, obesity, chronic illness, or clinical features suggestive of deficiency. Strongly agree: 39%, Agree: 57%, Neither agree nor disagree: 4%	96%
Preventive and Therapeutic Supplementation Strategy		
13	Preventive supplementation should align with age-specific recommended dietary intake and local dietary patterns. Strongly agree: 61%, Agree: 35%, Neither agree nor disagree: 4%	96%
14	Therapeutic supplementation should be individualized, guided by documented deficiency, clinical assessment, and appropriate follow-up. Strongly agree: 78%, Agree: 22%	100%
15	Adequate vitamin D status is necessary to optimize calcium and phosphorus absorption and support effective skeletal mineralization. Strongly agree: 78%, Agree: 22%	100%
16	Routine vitamin D supplementation is recommended during infancy to prevent deficiency-related bone disease. Strongly agree: 78%, Agree: 22%	100%
17	Beyond infancy, vitamin D supplementation should be considered in children with identifiable risk factors or inadequate sun exposure. Strongly agree: 48%, Agree: 48%, Neither agree nor disagree: 4%	96%
Environmental and Public Health Context		
18	Environmental and dietary factors, including soil mineral depletion, high phytate intake, and widespread use of demineralized water sources, may contribute to population-level mineral inadequacies affecting pediatric bone health. Strongly agree: 65%, Agree: 35%	100%
Formulation and Implementation Considerations		
19	The choice of mineral salt forms influences bioavailability, gastrointestinal tolerability, and overall clinical effectiveness in pediatric supplementation. Strongly agree: 70%, Agree: 26%, Neither agree nor disagree: 4%	96%
20	Age-appropriate and palatable dosage forms, including liquid preparations where suitable, can enhance adherence and thereby improve the clinical effectiveness of multi-mineral supplementation in children. Strongly agree: 61%, Agree: 39%	100%

MOSAIC-PBH framework

We propose a framework for PBH based on risk stratification (Figure 1). Children and adolescents should be assessed for clinical, nutritional, and environmental risk factors. In those at low risk, routine biochemical screening is generally not required; management may focus on ensuring adequate calcium and vitamin D intake, a balanced diet, sunlight exposure, and regular physical activity, with periodic reassessment. Children identified as at risk should undergo targeted clinical, dietary, and biochemical evaluation. Management may be guided by the presence of a deficiency or identified risk factors or clinical impairment. Preventive measures should be continued in those without deficiency, while affected individuals may receive nutritional supplementation, including calcium and vitamin D, with consideration of additional micronutrients such as magnesium and zinc when dietary inadequacy or deficiency is identified or suspected, along with lifestyle optimization and management of underlying conditions. Ongoing monitoring should be undertaken to assess response and guide treatment adjustments. This framework emphasizes early identification and a context-specific, multi-nutrient approach to support adequate bone accrual and long-term skeletal health.

**Figure 1:**
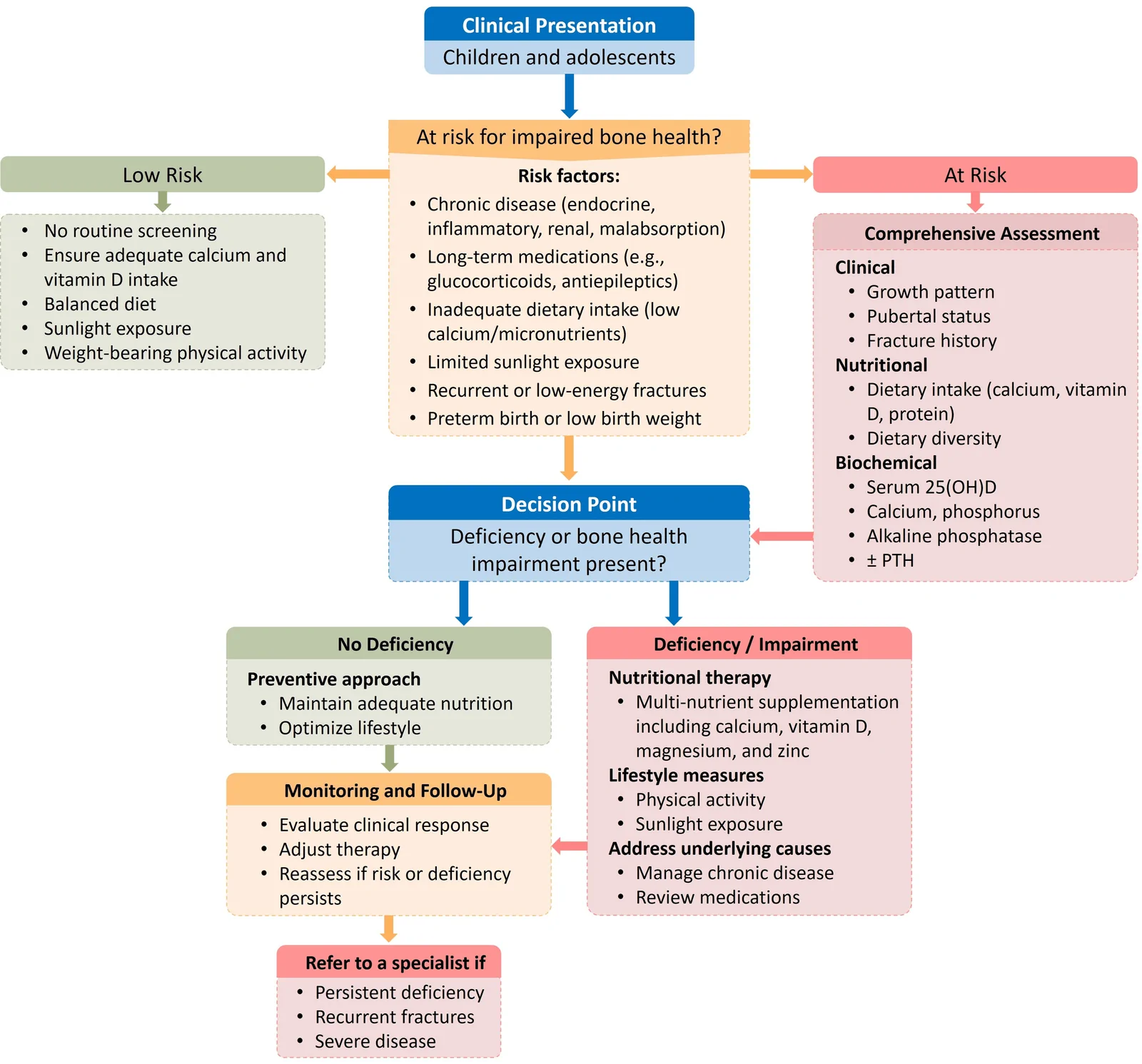
Proposed clinical pathway for pediatric bone health management: MOSAIC-PBH Framework MOSAIC-PBH: Multi-nutrient Optimization for Skeletal Advancement and Integrated Care in Pediatric Bone Health

## Conclusions

PBH is a multifactorial, systems-driven process shaped by interactions among nutrients, hormonal regulators, and environmental factors. This consensus highlights the potential for coexisting micronutrient inadequacies, including calcium, vitamin D, magnesium, and zinc, and supports consideration of a physiologically aligned multi-nutrient and multi-mineral framework alongside assessment and management of individual nutrient deficiencies during critical periods of growth.

Improving PBH requires risk-based screening, multidimensional assessment, and individualized preventive and therapeutic strategies, with particular emphasis on early life and adolescence. At the population level, especially in settings undergoing nutritional transition such as India, addressing hidden hunger requires nutrient-dense diets, greater dietary diversity, and context-specific approaches to supplementation. Future efforts should focus on generating high-quality evidence for multi-nutrient interventions and developing standardized, context-specific clinical guidelines.

## References

[1] Rodrick E, Kindler JM (2024). Bone mass accrual in children. Curr Opin Endocrinol Diabetes Obes.

[2] Bonjour JP, Theintz G, Law F, Slosman D, Rizzoli R (1994). Peak bone mass. Osteoporos Int.

[3] Baptista F, Baronio F (2025). Editorial: Bone health and development in children and adolescents, volume II. Front Endocrinol (Lausanne).

[4] Madhuchani D, Seneviratne SN, Ward LM (2023). Bone health in childhood and adolescence: an overview on dual-energy X-ray absorptiometry scanning, fracture surveillance and bisphosphonate therapy for low-middle-income countries. Front Endocrinol (Lausanne).

[5] Clark EM, Tobias JH, Ness AR (2006). Association between bone density and fractures in children: a systematic review and meta-analysis. Pediatrics.

[6] Bachrach LK (2005). Osteoporosis and measurement of bone mass in children and adolescents. Endocrinol Metab Clin North Am.

[7] Gordon RJ, Misra M, Mitchell DM (2000). Osteoporosis and bone fragility in children. Endotext [Internet].

[8] Guyot M, Ross W, Baez C, O'Brien M, McQuerry J (2026). Low vitamin D levels are associated with longer healing times in pediatric fracture patients. J Pediatr Soc North Am.

[9] Golden NH, Abrams SA (2014). Optimizing bone health in children and adolescents. Pediatrics.

[10] Moon RJ, Harvey NC, Davies JH, Cooper C (2014). Vitamin D and skeletal health in infancy and childhood. Osteoporos Int.

[11] Bouftas F, DeVries C (2024). Vitamin D and pediatric bone health: Important information and considerations for the pediatric orthopaedic surgeon. J Pediatr Soc North Am.

[12] Winzenberg T, Powell S, Shaw KA, Jones G (2011). Effects of vitamin D supplementation on bone density in healthy children: systematic review and meta-analysis. BMJ.

[13] Abrams SA (2021). Bone health in school age children: effects of nutritional intake on outcomes. Front Nutr.

[14] Gattrell WT, Logullo P, van Zuuren EJ (2024). ACCORD (ACcurate COnsensus Reporting Document): a reporting guideline for consensus methods in biomedicine developed via a modified Delphi. PLoS Med.

[15] Weffort VR, Lamounier JA (2024). Hidden hunger - a narrative review. J Pediatr (Rio J).

[16] Prentice A, Schoenmakers I, Laskey MA, de Bono S, Ginty F, Goldberg GR (2006). Nutrition and bone growth and development. Proc Nutr Soc.

[17] Peng L, Zhao J, Duan C, Yan J, Li D, Yang Y, He D (2025). Effects of vitamin A and vitamin D(3) supplementation on child growth and development in low- and middle-income countries: a systematic review and meta-analysis. Transl Pediatr.

[18] Awasthi S, Kumar D, Mahdi AA (2022). Prevalence of specific micronutrient deficiencies in urban school going children and adolescence of India: a multicenter cross-sectional study. PLoS One.

[19] Awasthi S, Kumar D, Dixit S (2023). Association of dietary intake with micronutrient deficiency in Indian school children: a cross-sectional study. J Nutr Sci.

[20] Harinarayan CV, Akhila H, Shanthisree E (2021). Modern India and dietary calcium deficiency-half a century nutrition data-retrospect-introspect and the road ahead. Front Endocrinol (Lausanne).

[21] (2026). Steady improvement in indicators for malnutrition [Internet]. [cited. Steady Improvement in Indicators for Malnutrition.

[22] Liao X, Chen S, Su M (2022). The relationship between dietary pattern and bone mass in school-age children. Nutrients.

[23] Beck JJ, Mahan ST, Nowicki P, Schreiber VM, Minkowitz B (2021). What is new in pediatric bone health. J Pediatr Orthop.

[24] Chacham S, Rajput S, Gurnurkar S (2020). Prevalence of vitamin D deficiency among infants in Northern India: a hospital based prospective study. Cureus.

[25] Mondkar S, Oza C, Dange N (2024). Assessment of vitamin D status, its determinants and relationship with bone health in indian children and young adults with type-1 diabetes. Indian J Endocrinol Metab.

[26] Stallings VA (1997). Calcium and bone health in children: a review. Am J Ther.

[27] Händel MN, Heitmann BL, Abrahamsen B (2015). Nutrient and food intakes in early life and risk of childhood fractures: a systematic review and meta-analysis. Am J Clin Nutr.

[28] Marles RJ (2017). Mineral nutrient composition of vegetables, fruits and grains: the context of reports of apparent historical declines. J Food Compos Anal.

[29] Rendu F, Peoc'h K, Berlin I, Thomas D, Launay JM (2011). Smoking related diseases: the central role of monoamine oxidase. Int J Environ Res Public Health.

[30] DiNicolantonio JJ, O'Keefe JH, Wilson W (2018). Subclinical magnesium deficiency: a principal driver of cardiovascular disease and a public health crisis. Open Heart.

[31] Ferguson EL, Gibson RS, Thompson LU, Ounpuu S (1989). Dietary calcium, phytate, and zinc intakes and the calcium, phytate, and zinc molar ratios of the diets of a selected group of East African children. Am J Clin Nutr.

[32] Kim OH, Booth CJ, Choi HS (2020). High-phytate/low-calcium diet is a risk factor for crystal nephropathies, renal phosphate wasting, and bone loss. Elife.

[33] Gibson RS, Bailey KB, Gibbs M, Ferguson EL (2010). A review of phytate, iron, zinc, and calcium concentrations in plant-based complementary foods used in low-income countries and implications for bioavailability. Food Nutr Bull.

[34] Proia P, Amato A, Drid P, Korovljev D, Vasto S, Baldassano S (2021). The Impact of diet and physical activity on bone health in children and adolescents. Front Endocrinol (Lausanne).

[35] Hansen M, Sandström B, Lönnerdal B (1996). The effect of casein phosphopeptides on zinc and calcium absorption from high phytate infant diets assessed in rat pups and Caco-2 cells. Pediatr Res.

[36] Surti A, Waheed H, Rehman R (2025). Environmental factors and vitamin D. The Impact of Vitamin D on Health and Disease: Beyond the Bones.

[37] Devulapalli CS (2025). Physical activity and vitamin D in children: a review of impacts on bone health and fitness. J Pediatr Endocrinol Metab.

[38] Maggioli C, Stagi S (2017). Bone modeling, remodeling, and skeletal health in children and adolescents: mineral accrual, assessment and treatment. Ann Pediatr Endocrinol Metab.

[39] Faienza MF, Urbano F, Chiarito M, Lassandro G, Giordano P (2023). Musculoskeletal health in children and adolescents. Front Pediatr.

[40] Siddiqui JA, Partridge NC (2016). Physiological bone remodeling: systemic regulation and growth factor involvement. Physiology (Bethesda).

[41] Stagi S, Cavalli L, Iurato C, Seminara S, Brandi ML, de Martino M (2013). Bone metabolism in children and adolescents: main characteristics of the determinants of peak bone mass. Clin Cases Miner Bone Metab.

[42] Hereford T, Kellish A, Samora JB, Reid Nichols L (2024). Understanding the importance of peak bone mass. J Pediatr Soc North Am.

[43] DeBoer MD, Weber DR, Zemel BS, Denburg MR, Herskovitz R, Long J, Leonard MB (2015). Bone mineral accrual is associated with parathyroid hormone and 1,25-dihydroxyvitamin D levels in children and adolescents. J Clin Endocrinol Metab.

[44] Levine MA (2012). Assessing bone health in children and adolescents. Indian J Endocrinol Metab.

[45] Pons-Belda OD, Alonso-Álvarez MA, González-Rodríguez JD, Mantecón-Fernández L, Santos-Rodríguez F (2023). Mineral metabolism in children: interrelation between vitamin D and FGF23. Int J Mol Sci.

[46] van den Hooven EH, Heppe DH, Kiefte-de Jong JC (2015). Infant dietary patterns and bone mass in childhood: the Generation R Study. Osteoporos Int.

[47] Joshi M, Uday S (2023). Vitamin D deficiency in chronic childhood disorders: importance of screening and prevention. Nutrients.

[48] Liu Y, Wang W, Yang Y, Deng J, Zhang Z (2025). Vitamin D and bone health: from physiological function to disease association. Nutr Metab (Lond).

[49] Shin YH, Shin HJ, Lee YJ (2013). Vitamin D status and childhood health. Korean J Pediatr.

[50] Kamilova AT, Alieva NR, Akhmedova DI, Abrorova BT (2025). Vitamin D and calcium-phosphorus in serum of children with celiac disease in a zone of high sunlight exposure. Front Pediatr.

[51] Rajab HA (2022). The effect of vitamin D level on parathyroid hormone and alkaline phosphatase. Diagnostics (Basel).

[52] Jakubowska-Pietkiewicz E (2024). Calcium-phosphate metabolism - selected disorders in children. Pediatr Endocrinol Diabetes Metab.

[53] Chanchlani R, Nemer P, Sinha R (2020). An overview of rickets in children. Kidney Int Rep.

[54] Lee JY, So TY, Thackray J (2013). A review on vitamin d deficiency treatment in pediatric patients. J Pediatr Pharmacol Ther.

[55] Escobedo-Monge MF, Bahillo-Curieses P, Parodi-Román J, Escobedo-Monge MA, Alonso-López P, Marugán-Miguelsanz JM (2024). Calcium, phosphate, and vitamin D in children and adolescents with chronic diseases: a cross-sectional study. Nutrients.

[56] Loughrill E, Wray D, Christides T, Zand N (2017). Calcium to phosphorus ratio, essential elements and vitamin D content of infant foods in the UK: possible implications for bone health. Matern Child Nutr.

[57] Ciosek Ż, Kot K, Kosik-Bogacka D, Łanocha-Arendarczyk N, Rotter I (2021). The effects of calcium, magnesium, phosphorus, fluoride, and lead on bone tissue. Biomolecules.

[58] Wu Z, Yuan Y, Tian J, Long F, Luo W (2021). The associations between serum trace elements and bone mineral density in children under 3 years of age. Sci Rep.

[59] Molenda M, Kolmas J (2023). The role of zinc in bone tissue health and regeneration-a review. Biol Trace Elem Res.

[60] Hong S (2025). Essential micronutrients in children and adolescents with a focus on growth and development: a narrative review. J Yeungnam Med Sci.

[61] Abrams SA, Atkinson SA (2003). Calcium, magnesium, phosphorus and vitamin D fortification of complementary foods. J Nutr.

[62] Koo WW (1988). Calcium, phosphorus, and vitamin D requirements of infants receiving parenteral nutrition. J Perinatol.

[63] Rigo J, Pieltain C, Salle B, Senterre J (2007). Enteral calcium, phosphate and vitamin D requirements and bone mineralization in preterm infants. Acta Paediatr.

[64] Jaisamrarn U, Esteban-Habana MA, Padolina CS (2023). Vitamins and minerals, education, and self-care need during preconception to 1000 days of life in Southeast Asia: an expert panel opinion. SAGE Open Med.

[65] Farias PM, Marcelino G, Santana LF (2020). Minerals in pregnancy and their impact on child growth and development. Molecules.

[66] Corsello A, Spolidoro GC, Milani GP, Agostoni C (2023). Vitamin D in pediatric age: current evidence, recommendations, and misunderstandings. Front Med (Lausanne).

[67] Rodd C, Kirouac N, Orkin J, Grimes R (2022). Evaluating and optimizing bone health in children with chronic health conditions. Paediatr Child Health.

[68] Kozgar SA, Chay P, Munns CF (2020). Screening of vitamin D and calcium concentrations in neonates of mothers at high risk of vitamin D deficiency. BMC Pediatr.

[69] Herdea A, Ionescu A, Dragomirescu MC, Ulici A (2023). Vitamin D-a risk factor for bone fractures in children: a population-based prospective case-control randomized cross-sectional study. Int J Environ Res Public Health.

[70] Bachrach LK, Sills IN (2011). Clinical report—bone densitometry in children and adolescents. Pediatrics.

[71] Ciancia S, van Rijn RR, Högler W, Appelman-Dijkstra NM, Boot AM, Sas TC, Renes JS (2022). Osteoporosis in children and adolescents: when to suspect and how to diagnose it. Eur J Pediatr.

[72] Jin HY, Noh ES, Hwang IT (2023). Diagnosis and management of osteoporosis in children and adolescents. Ewha Med J.

[73] Ekbote V, Khadilkar A, Chiplonkar S, Mughal Z, Khadilkar V (2013). Enhanced effect of zinc and calcium supplementation on bone status in growth hormone-deficient children treated with growth hormone: a pilot randomized controlled trial. Endocrine.

[74] Bacchetta J, Edouard T, Laverny G (2022). Vitamin D and calcium intakes in general pediatric populations: a French expert consensus paper. Arch Pediatr.

[75] Tonny RT, Roy RR, Jesmin T, Al Mamun A, Sultana N (2025). Management of rickets: the new horizons for the pediatrician. J Health Popul Nutr.

[76] Karimian P, Ebrahimi HK, Jafarnejad S, Delavar MA (2022). Effects of vitamin D on bone density in healthy children: a systematic review. J Family Med Prim Care.

[77] Chibuzor MT, Graham-Kalio D, Osaji JO, Meremikwu MM (2020). Vitamin D, calcium or a combination of vitamin D and calcium for the treatment of nutritional rickets in children. Cochrane Database Syst Rev.

[78] Rusińska A, Płudowski P, Walczak M (2018). Vitamin D supplementation guidelines for general population and groups at risk of vitamin D deficiency in Poland-recommendations of the Polish Society of Pediatric Endocrinology and Diabetes and the expert panel with participation of national specialist consultants and representatives of scientific societies-2018 update. Front Endocrinol (Lausanne).

[79] Holick MF, Binkley NC, Bischoff-Ferrari HA (2011). Evaluation, treatment, and prevention of vitamin D deficiency: an Endocrine Society clinical practice guideline. J Clin Endocrinol Metab.

[80] Amrein K, Scherkl M, Hoffmann M (2020). Vitamin D deficiency 2.0: an update on the current status worldwide. Eur J Clin Nutr.

[81] Choi J, Choe Y, Yang S (2023). Monthly versus daily administration of vitamin D3 in children: a retrospective propensity score-matched study. Front Endocrinol (Lausanne).

[82] (2013). Recommended vitamin D intake and management of low vitamin D status in adolescents: a position statement of the society for adolescent health and medicine. J Adolesc Health.

[83] Veith R, Holick (2018). The IOM-Endocrine Society controversy on recommended vitamin D targets: in support of the Endocrine Society position. Vitamin D (Fourth Edition).

[84] Nwosu BU (2025). Vitamin D for the prevention of diseases in children: a rebuttal to the 2024 Endocrine Society Clinical Practice Guideline. Front Endocrinol (Lausanne).

[85] Zhao J, Liu M, Yu D, Zhang Y (2024). Serum calcium and mortality in pediatric pneumonia patients admitted to the PICU: a retrospective cohort study. Sci Rep.

[86] Escobedo-Monge MF, Torres-Hinojal MC, Barrado E, Escobedo-Monge MA, Marugán-Miguelsanz JM (2021). Zinc nutritional status in a series of children with chronic diseases: a cross-sectional study. Nutrients.

[87] Ng DK, Ho RS, Wong AY, Leung SY (2025). Vitamin D and zinc deficiency in a pediatric population: a retrospective study. Pediatr Respirol Crit Care Med.

[88] Cao S, Hodges JK, McCabe LD, Weaver CM (2019). Magnesium requirements in children: recommendations for reevaluation and comparison with current evidence for adults. Nutrition Today.

[89] Escobedo-Monge MF, Barrado E, Parodi-Román J, Escobedo-Monge MA, Torres-Hinojal MC, Marugán-Miguelsanz JM (2022). Magnesium status and Ca/Mg ratios in a series of children and adolescents with chronic diseases. Nutrients.

[90] Liu J, Yuan E, Zhang Z, Jia L, Yin Z, Meng X, Du H (2012). Age- and sex-specific reference intervals for blood copper, zinc, calcium, magnesium, iron, lead, and cadmium in infants and children. Clin Biochem.

[91] Mihatsch W, Thome U, Saenz de Pipaon M (2021). Update on calcium and phosphorus requirements of preterm infants and recommendations for enteral mineral intake. Nutrients.

[92] (2020). Nutrient Requirements for Indians: Recommended Dietary Allowances (RDA) and Estimated Average Requirements (EAR). https://www.nin.res.in/RDA_Full_Report_2024.html.

[93] Binkovitz LA, Henwood MJ (2007). Pediatric DXA: technique and interpretation. Pediatr Radiol.

[94] Richards JD, Cori H, Rahn M (2025). Micronutrient bioavailability: concepts, influencing factors, and strategies for improvement. Front Nutr.

[95] (2026). National Institutes of Health: Magnesium - health professional fact sheet. https://ods.od.nih.gov/factsheets/Magnesium-HealthProfessional/.

[96] Devarshi PP, Mao Q, Grant RW, Hazels Mitmesser S (2024). Comparative absorption and bioavailability of various chemical forms of zinc in humans: a narrative review. Nutrients.

[97] Ceballos-Rasgado M, Lowe NM, Moran VH (2023). Toward revising dietary zinc recommendations for children aged 0 to 3 years: a systematic review and meta-analysis of zinc absorption, excretion, and requirements for growth. Nutr Rev.

[98] Bertinato J, Griffin P, Huliganga E, Matias FM, Dam D, Brooks SP (2020). Calcium exacerbates the inhibitory effects of phytic acid on zinc bioavailability in rats. J Trace Elem Med Biol.

[99] (2018). Applying methodological approaches to nutrient reference values for young children and women of reproductive age: an assessment of exemplar nutrients. Harmonization of Approaches to Nutrient Reference Values: Applications to Young Children and Women of Reproductive Age.

[100] Ternik R, Liu F, Bartlett JA (2018). Assessment of swallowability and palatability of oral dosage forms in children: report from an M-CERSI pediatric formulation workshop. Int J Pharm.

[101] Lopalco A, Denora N (2020). Paediatric formulation: design and development. Int J Mol Sci.

[102] Abrams SA (2010). Calcium absorption in infants and small children: methods of determination and recent findings. Nutrients.

[103] Silva TD, Vieira GM, Fraga LT, Fernandes WD, Sakane EN, Maeda SS (2025). Supplements for bone health. Arch Endocrinol Metab.

[104] Wu F, Fuleihan GE, Cai G (2023). Vitamin D supplementation for improving bone density in vitamin D-deficient children and adolescents: systematic review and individual participant data meta-analysis of randomized controlled trials. Am J Clin Nutr.

[105] Bahinipati J, Mishra A, Parida P, Tripathy R, Mohakud NK (2025). Evaluating vitamin D deficiency and toxicity in indian children: a retrospective study. Cureus.

[106] Rondanelli M, Faliva MA, Tartara A (2021). An update on magnesium and bone health. Biometals.

